# Association between region-specific epicardial adipose tissue and CT-derived fractional flow reserve-defined myocardial ischemia: a retrospective coronary CTA study stratified by hypertension and type 2 diabetes mellitus

**DOI:** 10.3389/fmed.2026.1883159

**Published:** 2026-06-17

**Authors:** Zheng Wang, Yunting Hu, Zuoqin Li, Xiang Wang

**Affiliations:** Department of Radiology, The Central Hospital of Wuhan, Tongji Medical College, Huazhong University of Science and Technology, Wuhan, China

**Keywords:** coronary CTA, CT-FFR, epicardial adipose tissue, hypertension, left atrioventricular groove, myocardial ischemia, type 2 diabetes mellitus

## Abstract

**Background:**

Epicardial adipose tissue (EAT) is a biologically active fat depot adjacent to the myocardium and coronary arteries and has been implicated in coronary atherosclerosis and myocardial ischemia. Although CT-derived fractional flow reserve (CT-FFR) provides integrated anatomic-functional assessment from coronary CTA, data on region-specific EAT phenotypes, particularly in patients with hypertension and type 2 diabetes mellitus (T2DM), remain limited.

**Purpose:**

To investigate the association between region-specific EAT thickness and CT-FFR-defined myocardial ischemia and to assess its independent and incremental value in patients stratified by hypertension and T2DM.

**Methods:**

In this retrospective study, consecutive individuals who underwent coronary CTA and CT-FFR analysis were included. Myocardial ischemia was defined as distal CT-FFR ≤ 0.80 in at least one major coronary artery. Region-specific EAT thicknesses were measured manually by two independent readers on standardized imaging planes. Multivariable logistic regression, ROC analysis, net reclassification improvement (NRI), integrated discrimination improvement (IDI), interaction analysis, and bootstrap internal validation were performed.

**Results:**

A total of 576 participants were analyzed, including 148 controls, 131 patients with hypertension only, 145 with T2DM only, and 152 with both conditions. Compared with controls, ischemic subgroups had greater EAT thickness in multiple regions, with the largest difference observed for left atrioventricular groove EAT (LAVG-EAT) (*p <* 0.001). In the fully adjusted model, LAVG-EAT remained independently associated with myocardial ischemia (OR 1.41, 95% CI 1.26–1.58; *p <* 0.001). LAVG-EAT yielded an AUC of 0.807, and its addition to the clinical plus coronary calcium burden model improved discrimination (AUC 0.886–0.912; DeLong *p =* 0.024), with a continuous NRI of 0.168 and an IDI of 0.023. The optimism-corrected AUC was 0.907.

**Conclusion:**

Region-specific EAT, particularly LAVG-EAT, was independently associated with CT-FFR-defined myocardial ischemia and provided incremental discriminatory value beyond conventional clinical variables and coronary calcium burden. LAVG-EAT may serve as a practical adjunctive imaging marker for ischemia identification and risk stratification in patients with hypertension and/or T2DM.

## 1 Introduction

Epicardial adipose tissue (EAT) is located between the myocardium and the visceral pericardium and closely follows the course of the coronary arteries, with minimal fascial separation. Beyond serving as an energy depot, EAT exhibits immunoinflammatory and metabolic endocrine properties and may influence coronary endothelial function, local inflammation, and fibrosis through paracrine/vasocrine signaling and microcirculatory pathways (1–5). Classic mechanistic evidence supports a local fat-vessel axis, whereby EAT-derived mediators contribute to perivascular inflammation and vascular remodeling (5).

CT-derived fractional flow reserve (CT-FFR) is a noninvasive functional metric derived from coronary CTA that can identify hemodynamically significant coronary lesions and has been incorporated into expert consensus statements as an important component of integrated anatomic-functional assessment (6). Meanwhile, a growing body of evidence has linked EAT imaging phenotypes to ischemia and prognosis, including improved prediction of lesion-specific ischemia when combined with plaque assessment, ischemia-related radiomics signatures, and deep-learning-based EAT quantification associated with future myocardial infarction risk (7–9).

However, two common gaps remain in the literature. First, most studies have focused on global EAT volume, attenuation, or radiomics, whereas systematic comparison of region-specific EAT thickness within the same cohort remains limited. Second, stratification according to high-risk comorbidity phenotypes such as hypertension and T2DM is still uncommon, restricting interpretation of how blood pressure load and insulin resistance may shape the spatial distribution of EAT and the risk of functional myocardial ischemia (10–12). We therefore conducted this retrospective study using standardized manual measurements of multiregional EAT thickness combined with a hypertension/T2DM stratification strategy to evaluate the independent and incremental value of region-specific EAT metrics in relation to functional myocardial ischemia.

## 2 Materials and methods

### 2.1 Study design and participants

This was a single-center retrospective study. We screened consecutive individuals who underwent coronary computed tomography angiography (CCTA) for chest pain or suspected coronary artery disease and completed CT-derived fractional flow reserve (CT-FFR) analysis between January 2022 and December 2025.

The inclusion criteria were: (1) age ≥18 years; (2) diagnostic-quality CCTA adequate for post-processing and CT-FFR computation; and (3) complete clinical information, including cardiovascular risk factor and metabolic profile data.

The exclusion criteria were: (1) prior coronary stent implantation or coronary artery bypass grafting; (2) prior myocardial infarction, severe cardiomyopathy, or significant valvular heart disease; (3) congenital heart disease or severe arrhythmia compromising image quality; (4) non-diagnostic CCTA precluding EAT or CT-FFR analysis; and (5) missing key variables.

### 2.2 CT-FFR-based definition of ischemia

Functional myocardial ischemia was defined as distal CT-FFR ≤ 0.80 in any major coronary artery [left main (LM), left anterior descending (LAD), left circumflex (LCX), or right coronary artery (RCA)]. Non-ischemia was defined as CT-FFR > 0.80.

Patient-level grouping was based on the lowest CT-FFR value among the major vessels (distal or lesion-distal, as applicable).

### 2.3 Control group and ischemic subgroups

Controls were defined as participants with CT-FFR > 0.80 and no evidence of functional ischemia. Participants in the control group were allowed to have hypertension and/or T2DM.

Patients meeting the ischemia criteria were further stratified by comorbidity phenotype into: (1) the HTN group, defined as hypertension without T2DM; (2) the T2DM group, defined as T2DM without hypertension; and (3) the HTN + T2DM group, defined as the coexistence of both hypertension and T2DM.

### 2.4 Definitions of hypertension and T2DM

Hypertension was determined retrospectively on the basis of a documented diagnosis or ongoing antihypertensive therapy, and/or repeated outpatient or inpatient blood pressure measurements within the hypertensive range (BP ≥ 140/90 mmHg) (11).

T2DM was determined retrospectively on the basis of a documented diagnosis or ongoing glucose-lowering therapy, and/or laboratory criteria meeting diagnostic thresholds, such as HbA1c ≥ 6.5%, fasting plasma glucose ≥7.0 mmol/L, or 2-h oral glucose tolerance test glucose ≥11.1 mmol/L (12).

### 2.5 CCTA acquisition protocol

All participants underwent CCTA on a 64-slice dual-source CT scanner (Somatom Definition Flash, Siemens Healthineers, Germany). The scan range covered the entire heart from below the carina to the diaphragm. A nonionic iodinated contrast agent (370 mgI/mL) was administered at 60–70 mL, followed by 30 mL saline at 5.0–5.5 mL/s. Bolus tracking was performed with a region of interest placed at the aortic root (trigger threshold 100 HU), and acquisition was initiated after an 8-s delay. Retrospective ECG gating was used. Typical parameters included a tube voltage of 100 kV, tube current of 280 mAs, reconstruction thickness of 0.75 mm, and a total acquisition time of approximately 4.0 s.

### 2.6 CT-FFR analysis and coronary artery calcium scoring

CT-FFR and the total coronary artery calcium (CAC) score (Agatston score) were automatically calculated from the raw CTA data using an AI-assisted one-stop CCTA platform (Shukun Technology, Beijing, China). The software first performed coronary segmentation and three-dimensional reconstruction and then calculated CT-FFR along the coronary tree using a computational fluid dynamics (CFD) model. Distal or lesion-distal CT-FFR values for the major vessels were recorded, and the minimum CT-FFR value was used for patient-level classification.

Post-processing was performed by experienced cardiovascular imaging physicians who were blinded to the EAT measurements and clinical grouping. Centerlines, stenosis localization, and CT-FFR outputs were reviewed and corrected when necessary. Interpretation of CT-FFR followed international expert consensus statements and domestic recommendations (6, 13).

The total CAC score was obtained using the standard Agatston method. In routine clinical workflow, CAC scoring could be based on an ECG-gated non-contrast scan acquired before CCTA or on a prior CAC examination obtained under comparable conditions. Automated calcium detection and scoring were reviewed by imaging physicians. Previous work has suggested good agreement between automated CAC assessment on this platform and standard reference methods for risk stratification (14).

### 2.7 Measurement of region-specific EAT thickness

**Figure 1:**
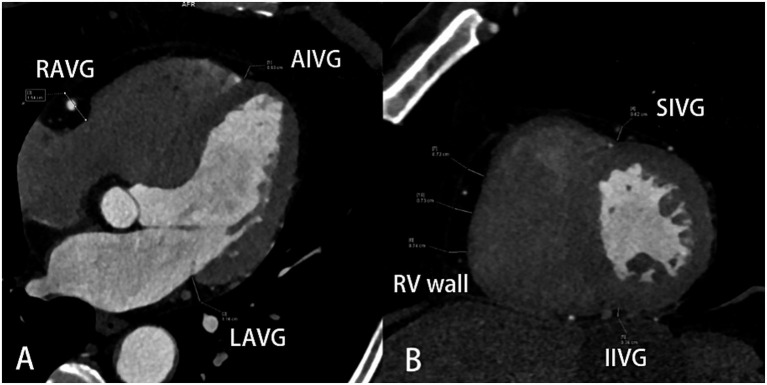
Measurement of EAT thickness at the RAVG, LAVG, and AIVG on the horizontal long-axis plane and at the SIVG, IIVG, and right ventricular free wall on the basal short-axis plane. RV free-wall EAT thickness was averaged from three equidistant points (25, 50, and 75%).

Measurement principles: EAT thickness was measured manually. According to standardized protocols from previous studies of region-specific EAT, all measurements were performed at a fixed reconstruction phase (e.g., 75% of the R-R interval) to reduce the influence of cardiac-cycle variability on thickness. Standardized horizontal long-axis and basal short-axis planes were selected. On the horizontal long-axis plane, EAT thickness was measured at the right atrioventricular groove (RAVG), left atrioventricular groove (LAVG), and anterior interventricular groove (AIVG). On the basal short-axis plane, EAT thickness was measured at the superior interventricular groove (SIVG) and inferior interventricular groove (IIVG). Right ventricular free-wall EAT thickness was measured at three equidistant points (25, 50, and 75%) along the RV wall on the basal short-axis plane, and the average was used (Figure 1). For each participant, all regional EAT thicknesses were independently measured by two observers, and the mean of the two measurements was used for the final analysis. Inter- and intra-observer agreement was assessed using ICCs for all regional EAT measurements, which are reported in Section 3.4.

### 2.8 Covariates

Demographic and clinical variables included age, sex, body mass index (BMI), smoking status, systolic blood pressure, LDL-C, HDL-C, and HOMA-IR. The total CAC score (Agatston score) was used as an anatomic burden indicator to control for the influence of calcified plaque burden on the classification of functional myocardial ischemia (6).

### 2.9 Statistical analysis

Continuous data are reported as mean ± standard deviation for normally distributed variables and as median (interquartile range) otherwise, whereas categorical variables are expressed as counts with percentages. Between-group differences were examined using one-way analysis of variance or the Kruskal-Wallis test for continuous variables and the chi-square test for categorical variables, as appropriate. To account for multiple comparisons across regional EAT measurements, false discovery rate adjustment was performed using the Benjamini-Hochberg procedure.

Logistic regression models were constructed with CT-FFR-defined myocardial ischemia (CT-FFR ≤ 0.80) as the dependent variable. Model 1 included age, sex, BMI, and smoking. Model 2 included Model 1 plus systolic blood pressure, LDL-C, HDL-C, and HOMA-IR. Model 3 included Model 2 plus total CAC score (Agatston). Model 4 included Model 3 plus LAVG-EAT.

Incremental value was assessed using ROC-AUC, DeLong testing, net reclassification improvement (NRI), and integrated discrimination improvement (IDI). Interaction and stratified analyses were performed to determine whether the association between LAVG-EAT and myocardial ischemia varied according to hypertension or T2DM status (interaction terms: LAVG-EAT × HTN; LAVG-EAT × T2DM). Variance inflation factors (VIFs) were used to assess multicollinearity. Bootstrap resampling (1,000 iterations) was used for internal validation, and the optimism-corrected AUC, calibration slope, Brier score, and coefficient stability were reported. All analyses were performed using SPSS version 26.0, and a two-sided *p <* 0.05 was considered statistically significant.

LAVG-EAT was pre-specified as the primary regional metric for multivariable modeling on the basis of its standardization, reproducibility, and biological rationale; because regional EAT measures are strongly inter-correlated, they were not entered simultaneously to avoid multicollinearity.

## 3 Results

### 3.1 Study cohort and baseline characteristics

A total of 576 participants were included, including 148 controls, 131 in the HTN-only group, 145 in the T2DM-only group, and 152 in the HTN + T2DM group. Baseline characteristics are summarized in Table 1. Significant between-group differences were observed in age, BMI, blood pressure, glucose and lipid metabolism indices, insulin resistance, total CAC score, and minimum CT-FFR, whereas sex distribution showed a borderline difference and smoking status did not differ significantly.

**Table 1:** Baseline clinical characteristics and major imaging parameters across groups.

Variable	Control (*n =* 148)	HTN group (*n =* 131)	T2DM group (*n =* 145)	HTN + T2DM group (*n =* 152)	*P*-value
Age (years)	62.2 ± 11.4	67.5 ± 10.2	67.1 ± 10.7	67.4 ± 8.6	<0.001
Male, *n* (%)	85 (57.4)	87 (66.4)	103 (71.0)	107 (70.4)	0.050
Hypertension, *n* (%)	67 (45.3)	131 (100)	0 (0)	152 (100)	–
Type 2 diabetes, *n* (%)	74 (50)	0 (0)	145 (100)	152 (100)	–
BMI (kg/m^2^)	23.8 ± 2.6	24.3 ± 2.6	24.6 ± 3.2	24.7 ± 3.0	0.032
Current smoker, *n* (%)	64 (43.2)	58 (44.3)	80 (55.2)	75 (49.3)	0.160
Systolic BP (mmHg)	136.9 ± 18.3	148.8 ± 21.3	134.2 ± 16.5	155.2 ± 23.2	<0.001
Diastolic BP (mmHg)	80.4 ± 11.2	84.1 ± 11.1	79.8 ± 10.5	86.0 ± 12.1	<0.001
Total cholesterol (mmol/L)	4.71 ± 0.94	4.67 ± 0.87	4.73 ± 0.87	5.01 ± 1.02	0.007
Triglycerides (mmol/L)	1.20 ± 0.39	1.06 ± 0.36	1.31 ± 0.46	1.55 ± 0.52	<0.001
LDL-C (mmol/L)	2.54 ± 0.82	2.64 ± 0.81	2.90 ± 0.81	2.71 ± 0.80	0.002
HDL-C (mmol/L)	1.08 ± 0.25	1.01 ± 0.23	0.97 ± 0.25	0.99 ± 0.26	0.001
Fasting plasma glucose (mmol/L)	6.27 ± 2.19	5.41 ± 0.68	8.78 ± 2.64	9.14 ± 2.77	<0.001
Fasting insulin (uIU/mL)	10.88 ± 4.31	11.98 ± 4.91	13.75 ± 5.06	15.55 ± 5.58	<0.001
HOMA-IR	2.74 ± 1.41	3.11 ± 1.30	5.48 ± 2.24	6.42 ± 2.61	<0.001
Total CAC score (Agatston)	74 (20–176)	166 (76–332)	193 (101–402)	271 (146–533)	<0.001
Minimum CT-FFR	0.86 ± 0.04	0.73 ± 0.05	0.69 ± 0.06	0.64 ± 0.08	<0.001

*In the control group, 26 (17.6%) had hypertension only, 33 (22.3%) had T2DM only, 41 (27.7%) had both, and 48 (32.4%) had neither.

### 3.2 CT-FFR values and coronary calcium burden

The lowest CT-FFR was 0.86 ± 0.04 in the control group and 0.73 ± 0.05, 0.69 ± 0.06, and 0.64 ± 0.08 in the HTN, T2DM, and HTN + T2DM groups, respectively (*p <* 0.001). The total CAC score showed a stepwise increase across the ischemic subgroups (*p <* 0.001; Table 1).

### 3.3 Comparison of region-specific EAT thickness

Compared with the control group, region-specific EAT thickness was increased in most ischemic subgroups. The most pronounced difference was observed for LAVG-EAT, which increased from 11.61 ± 3.18 mm in controls to 14.21 ± 3.27, 15.43 ± 3.35, and 16.27 ± 3.63 mm in the HTN, T2DM, and HTN + T2DM groups, respectively (*p <* 0.001). Other regional measures, including RV free-wall EAT, AIVG-EAT, and SIVG-EAT, also showed significant between-group differences. No significant between-group differences were observed for RAVG-EAT or IIVG-EAT. The complete regional EAT thicknesses for all groups, with raw and Benjamini-Hochberg–adjusted *p*-values, are presented in Table 2.

**Table 2:** Region-specific EAT thickness across groups (mm).

Region	Control (*n =* 148)	HTN (*n =* 131)	T2DM (*n =* 145)	HTN + T2DM (*n =* 152)	*P* (raw)	*P* (BH-adjusted)
RAVG-EAT	13.62 ± 3.58	13.49 ± 3.71	13.95 ± 3.63	13.78 ± 3.69	0.612	0.734
LAVG-EAT	11.61 ± 3.18	14.21 ± 3.27	15.43 ± 3.35	16.27 ± 3.63	<0.001	<0.001
AIVG-EAT	8.34 ± 2.62	9.45 ± 2.71	9.88 ± 2.84	10.42 ± 2.93	<0.001	<0.001
SIVG-EAT	6.71 ± 2.18	7.62 ± 2.25	8.05 ± 2.34	8.43 ± 2.41	<0.001	0.001
IIVG-EAT	6.92 ± 2.41	7.05 ± 2.33	7.21 ± 2.52	7.13 ± 2.45	0.812	0.812
RV free-wall EAT	4.88 ± 1.72	5.61 ± 1.79	5.94 ± 1.88	6.32 ± 1.95	<0.001	0.001

### 3.4 Reproducibility

Inter- and intra-observer agreement was good to excellent for all regional EAT measurements. The inter-observer ICCs were 0.82 (RAVG), 0.87 (LAVG), 0.85 (AIVG), 0.83 (SIVG), 0.80 (IIVG), and 0.79 (RV free-wall), and the corresponding intra-observer ICCs were 0.86, 0.92, 0.89, 0.88, 0.85, and 0.84, respectively. LAVG-EAT showed the highest reproducibility among all regions, supporting its use as the primary regional metric in the main analyses.

### 3.5 Logistic regression and independent association

**Figure 2:**
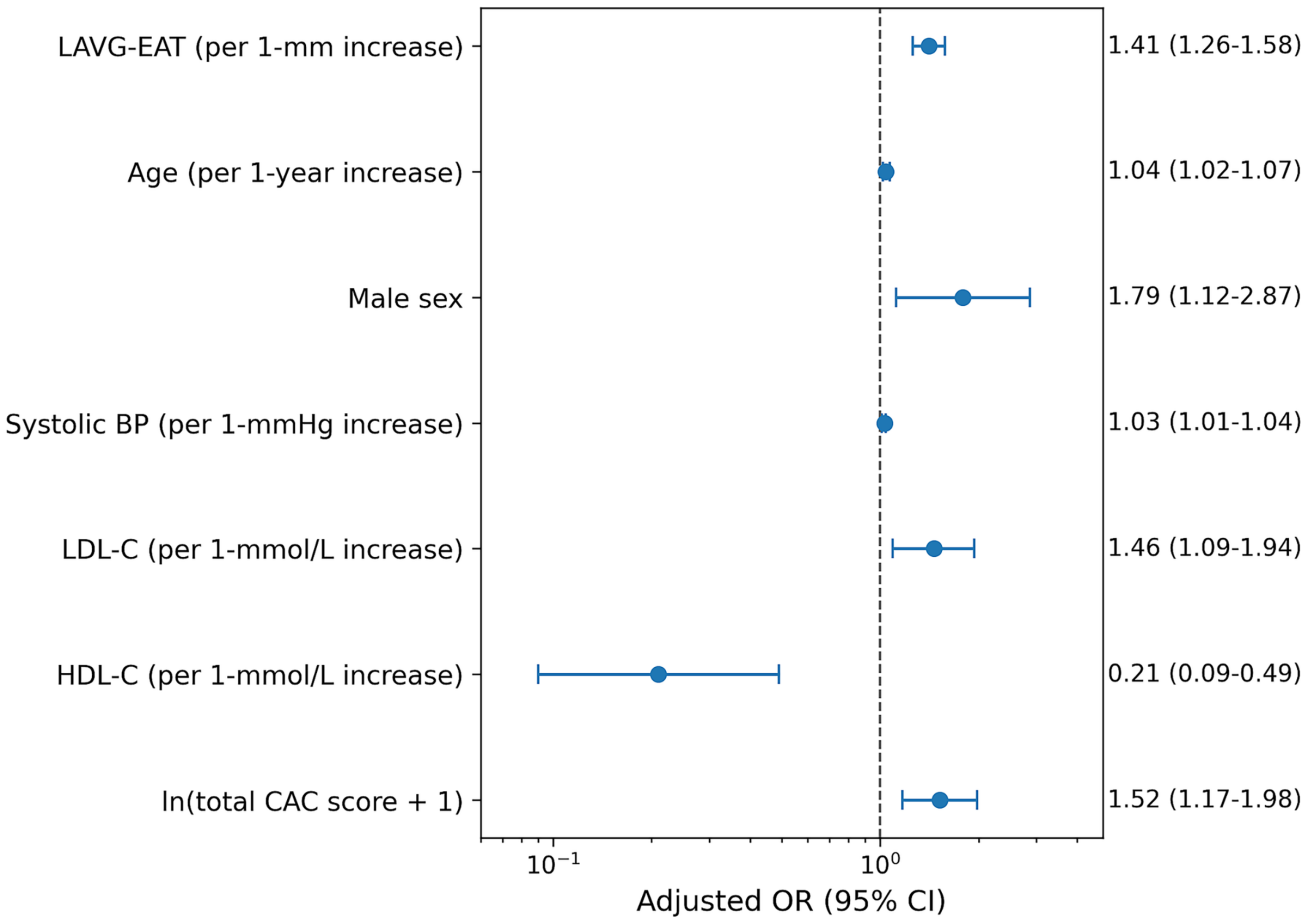
Forest plot of independent predictors of CT-derived fractional flow reserve-defined myocardial ischemia in the fully adjusted model.

Univariable logistic regression showed that LAVG-EAT was significantly associated with myocardial ischemia (OR 1.38, 95% CI 1.30–1.46; *p <* 0.001). This association remained significant after multivariable adjustment for conventional risk factors, metabolic variables, and total CAC score, with an adjusted OR of 1.41 (95% CI 1.26–1.58; *p <* 0.001). The remaining independent predictors are presented in Figure 2.

### 3.6 Correlation analysis

LAVG-EAT thickness was positively correlated with HOMA-IR (r = 0.44), systolic blood pressure (r = 0.40), and total CAC score (r = 0.49), and negatively correlated with the minimum CT-FFR (r = −0.50; all *p <* 0.001). In addition, RV free-wall EAT showed a closer association with blood pressure parameters, being positively correlated with systolic blood pressure (r = 0.29, *p <* 0.001) and diastolic blood pressure (r = 0.21, *p =* 0.002), whereas no similarly strong correlations were observed for HOMA-IR or minimum CT-FFR.

### 3.7 Discriminative performance, interaction analysis, and internal validation

LAVG-EAT showed moderate discriminatory ability for CT-FFR-defined myocardial ischemia (AUC 0.807, 95% CI 0.768–0.844), with an optimal cutoff of 12.86 mm. Adding LAVG-EAT to the clinical plus coronary calcium burden model improved discrimination (AUC 0.886–0.912; DeLong *p =* 0.024) and risk reclassification (continuous NRI 0.168; IDI 0.023). Bootstrap internal validation yielded an optimism-corrected AUC of 0.907, with acceptable calibration and no evidence of marked multicollinearity. The association between LAVG-EAT and myocardial ischemia appeared stronger in the T2DM subgroup, whereas no significant interaction was observed with hypertension. The detailed results of discrimination, reclassification, interaction analysis, stratified analysis, and internal validation are summarized in Table 3.

**Table 3:** Discriminative performance, reclassification, interaction/stratified analysis, and internal validation results for LAVG-EAT.

Metric	Value
AUC of LAVG-EAT (95% CI)	0.807 (0.768–0.844)
Optimal cutoff (mm)	12.86
Model AUC before adding LAVG-EAT	0.886
Model AUC after adding LAVG-EAT	0.912
DeltaAUC/DeLong P	0.026/0.024
Continuous NRI (95% CI)	0.168 (0.061–0.275); *P =* 0.002
IDI (95% CI)	0.023 (0.010–0.039); *P =* 0.001
Bootstrap optimism-corrected AUC	0.907
Calibration slope	0.94
Brier score	0.121
VIF range	1.16–2.86
Adjusted OR in T2DM subgroup (per 1-mm increase)	1.46 (1.30–1.64)
Adjusted OR in HTN subgroup (per 1-mm increase)	1.37 (1.22–1.54)
Mean bootstrap SD of the LAVG-EAT coefficient	0.08
Main interaction findings	LAVG-EAT × T2DM significant (*p =* 0.032); LAVG-EAT × HTN not significant (*p =* 0.118)

## 4 Discussion

This study showed that, in a retrospective CCTA/CT-FFR cohort stratified by hypertension and T2DM, region-specific EAT thickness was significantly associated with CT-FFR-defined functional myocardial ischemia, with LAVG-EAT emerging as the most prominent regional marker in the present analysis. The association remained significant after adjustment for conventional cardiovascular risk factors, metabolic parameters, and coronary calcium burden (Agatston score). Beyond statistical significance, incorporation of LAVG-EAT into the clinical plus coronary calcium burden model provided measurable incremental discrimination, and this added value was supported by NRI/IDI and bootstrap internal validation. These findings suggest that, compared with global EAT measures, region-specific EAT phenotypes anchored to specific anatomic sites may more closely reflect the local perivascular microenvironment associated with hemodynamically relevant coronary disease.

We observed a moderately strong positive correlation between total CAC (Agatston score) and LAVG-EAT (r = 0.49), which is consistent with the hypothesis that these two markers may share upstream determinants such as aging, adiposity, insulin resistance, and chronic low-grade inflammation/oxidative stress (15). More importantly, LAVG-EAT remained significantly associated with myocardial ischemia even after adjustment for CAC, indicating that local adipose phenotyping may provide information that cannot be fully attributed to calcified plaque burden alone. This is consistent with previous findings in hypertensive populations showing that EAT is associated with coronary atherosclerotic burden and CT-FFR-related functional endpoints (16).

The correlation patterns observed in the present study also suggest regional heterogeneity. LAVG-EAT was more strongly associated with metabolic and insulin-resistance markers such as HOMA-IR, whereas RV free-wall EAT showed more modest but directionally consistent correlations with systolic and diastolic blood pressure, supporting a closer relationship with blood pressure-related parameters. Such heterogeneity may reflect differences in coronary adjacency, local mechanical stress, microvascular perfusion, and regional adipose biology, including developmental origin and neurovascular distribution. Recent CMR studies have suggested that atrioventricular groove adipose tissue is easier to standardize and tends to be increased in metabolic syndrome and T2DM (17). CT-based studies have also linked EAT abnormalities in T2DM to early structural and functional cardiac alterations (18). Hypertension has been associated with fat deposition, inflammation, and fibrosis in the setting of chronic pressure overload and sympathetic activation, and may in turn be related to coronary vasomotion and microcirculatory function through perivascular signaling pathways (2–4, 16). In T2DM, insulin resistance and systemic low-grade inflammation have been associated with adipose dysfunction and microvascular abnormalities, which may provide biological plausibility for the observed association between cardiometabolic phenotype, local EAT expansion, and myocardial ischemia (2–5, 19). The interaction analysis further suggested that the association between LAVG-EAT and myocardial ischemia may be stronger in the T2DM subgroup.

The clinical relevance of the present findings does not lie in replacing CT-FFR with region-specific EAT. CT-FFR remains a direct functional indicator of myocardial ischemia. Rather, region-specific EAT can be obtained directly from routine CCTA images without additional functional post-processing. In clinical practice, it may therefore serve as an adjunctive imaging marker for identifying patients who warrant further functional assessment or more intensive integrated cardiometabolic management. Region-specific EAT rather than pericoronary adipose tissue was selected in this study mainly because the analysis was based on retrospective routine CCTA data, in which standardized regional thickness measurements were easier to implement across the full cohort and showed better reproducibility. By contrast, pericoronary adipose tissue is more lesion-specific and more closely related to the pathophysiology of individual plaques, but it usually requires more dedicated vessel-level analysis (20). Accordingly, the present study used region-specific EAT as a more pragmatic imaging phenotype rather than as a substitute for lesion-level adipose assessment.

Several mechanistic aspects of this association deserve careful consideration. Given the cross-sectional and retrospective nature of the study, the temporal relationship between regional EAT expansion and myocardial ischemia cannot be determined. Accordingly, the observed association should not be interpreted as evidence of causality. Importantly, the relationship may be reverse or bidirectional. While expanded EAT has been proposed as a contributor to ischemia through local inflammatory and metabolic effects, it is also plausible that chronic myocardial ischemia creates a pro-inflammatory and hypoxic microenvironment that promotes adaptive changes in adjacent perivascular and epicardial adipose tissue. Through paracrine and vasocrine pathways, ischemic myocardial tissue may facilitate local adipose accumulation, phenotypic remodeling, or both. In addition, the association may reflect the influence of common upstream factors rather than a direct causal link. Aging, overall adiposity, insulin resistance, and chronic low-grade systemic inflammation are all capable of promoting both EAT expansion and coronary physiological impairment, potentially generating the cross-sectional correlation observed in this study. These possibilities cannot be disentangled in the present design and can only be clarified by longitudinal studies that track temporal changes in regional EAT and CT-FFR.

Our findings can be compared with the recent CCTA study by Gül et al. (21), which evaluated epicardial fat volume (EFV), epicardial fat attenuation, and pericoronary fat tissue thickness (PCFT) in relation to coronary calcium burden in 255 patients. In that study, PCFT around the right coronary artery (RCA), located in the right atrioventricular groove, was the strongest pericoronary fat marker. RCA-PCFT independently predicted coronary calcium burden (OR 1.19, *p =* 0.001) and showed the highest discriminative performance (AUC 0.718), even exceeding EFV. In line with these findings, LAVG-EAT was the strongest regional adipose marker of CT-FFR-defined ischemia in our cohort. Although the study populations and endpoints were different, both studies identified atrioventricular-groove fat as the most informative regional fat depot. Notably, the optimal LAVG-EAT cutoff in our study (12.86 mm) was very close to the PCFT threshold for obstructive coronary disease (12.87 mm) reported in the literature cited by Gül et al. (21). Importantly, our endpoint was CT-FFR-defined ischemia rather than coronary calcium burden. While Gül et al. (21) found an association between fat metrics and coronary calcium, LAVG-EAT in our study remained associated with ischemia after adjustment for calcium burden and improved risk discrimination as shown by NRI and IDI analyses. In addition, LAVG-EAT increased progressively across hypertension and T2DM strata. These findings suggest that atrioventricular-groove fat may provide information beyond coronary calcification alone, and that its relevance is closely tied to the underlying metabolic milieu. We did not evaluate epicardial fat attenuation, which was included in the study by Gül et al. (21); future studies combining regional fat thickness and attenuation may provide a more comprehensive assessment of epicardial adipose tissue.

Building on this metabolic link, our findings may be particularly relevant to patients with metabolic dysfunction-associated steatotic liver disease (MASLD), a cardiometabolic condition characterized by insulin resistance, chronic low-grade inflammation, visceral adiposity, and increased epicardial adipose tissue accumulation. Previous studies have shown that MASLD is associated with early myocardial dysfunction, including impaired left ventricular global longitudinal strain, even in the absence of overt cardiovascular disease (22). Consistent with such a metabolic substrate, LAVG-EAT in our cohort showed the strongest correlation with HOMA-IR while remaining independently associated with CT-FFR-defined ischemia, suggesting that regional EAT may link systemic metabolic abnormalities to coronary functional impairment. Because EAT can be measured directly from routine CCTA, regional EAT assessment may provide additional information for cardiovascular risk evaluation in patients with MASLD. However, hepatic steatosis was not systematically assessed in our cohort, and no direct conclusions regarding MASLD can be drawn from the present data. Future prospective studies in well-characterized MASLD populations are needed to determine whether regional EAT measurements are associated with ischemic risk and cardiovascular outcomes.

### 4.1 Limitations

This study has several limitations. First, the single-center, cross-sectional, retrospective design precludes causal inference and cannot determine the direction of the EAT–ischemia association; reverse or bidirectional relationships (e.g., reactive EAT expansion secondary to chronic ischemia-related inflammation) cannot be excluded, and the cohort remains susceptible to selection bias. Second, EAT thickness was measured manually; although reproducibility was good, reader-related variability cannot be completely eliminated. Third, EAT thickness is only a morphologic surrogate and does not directly reflect the biological activity of adipose tissue. Fourth, residual confounding is still possible, particularly because information on physical activity, diet, disease duration, and medication adherence was not fully available. Fifth, stenosis severity and plaque composition were not consistently archived across the full cohort and therefore were not incorporated into the multivariable model. Finally, although the VIF and bootstrap results were relatively stable, the possibility of overfitting cannot be fully excluded. Future studies should integrate multidimensional adipose phenotypes, lesion-level plaque characteristics, and validation in prospective multicenter cohorts.

### 4.2 Future directions

Prospective multicenter studies using standardized measurement protocols are needed to validate the proposed cutoff value and to further define the clinical utility of region-specific EAT. Longitudinal follow-up and interventional studies may also help determine whether changes in regional EAT are accompanied by improvement in CT-FFR, progression of CAC, and changes in hard clinical outcomes.

## 5 Conclusion

Within an integrated coronary CTA and CT-FFR assessment framework, region-specific EAT was independently associated with CT-FFR-defined functional myocardial ischemia, with LAVG-EAT emerging as the most prominent regional marker in the present analysis. After adjustment for conventional risk factors and coronary calcium burden, LAVG-EAT retained incremental discriminative value and may provide useful complementary information for ischemia risk identification, further functional assessment, and integrated risk stratification in patients with hypertension and/or type 2 diabetes mellitus.

## Data Availability

The raw data supporting the conclusions of this article will be made available by the authors, without undue reservation.
